# Controlling Noncommunicable Diseases in Transitional Economies: Mental Illness in Suicide Attempters in Singapore—An Exploratory Analysis

**DOI:** 10.1155/2019/4652846

**Published:** 2019-01-15

**Authors:** Carol C. Choo, Peter K. H. Chew, Roger C. Ho

**Affiliations:** ^1^College of Healthcare Sciences, James Cook University, 387380, Singapore; ^2^Department of Psychological Medicine, National University of Singapore, 119228, Singapore; ^3^Centre of Excellence in Behavioral Medicine, Nguyen Tat Thanh University (NTTU), Ho Chi Minh City, 70000, Vietnam; ^4^Faculty of Education, Huaibei Normal University, 100 Dongshan Road, Huaibei, Anhui 235000, China; ^5^Biomedical Global Institute of Healthcare Research & Technology (BIGHEART), Yong Loo Lin School of Medicine, National University of Singapore, Singapore 117599, Singapore

## Abstract

**Background:**

Mental illness is a pertinent risk factor related to suicide. However, research indicates there might be underdiagnosis of mental illness in Asian suicide attempters; this phenomenon is concerning. This study explored prediction of diagnosis of mental illness in suicide attempters in Singapore using available variables.

**Methods:**

Three years of medical records related to suicide attempters (*N* = 462) who were admitted to the emergency department of a large teaching hospital in Singapore were subjected to analysis. Of the sample, 25% were diagnosed with mental illness; 70.6% were females and 29.4% were males; 62.6% were Chinese, 15.4% Malays, and 16.0% Indians. Their age ranged from 12 to 86 (*M* = 29.37,* SD* = 12.89). All available variables were subjected to regression analyses.

**Findings:**

The full model was significant in predicting cases with and without diagnosis of mental illness and accurately classified 79% of suicide attempters with diagnosis of mental illness.

**Conclusions:**

The findings were discussed in regard to clinical implications in diagnosis and primary prevention.

## 1. Introduction

Suicide has become a serious problem worldwide [1]. Suicide attempts are also a serious public health problem, with significant tolls for psychiatric and other healthcare services [2]. Multidimensional psychosocial factors were related to suicide deaths, e.g., past suicide attempts, adverse life events, substance and alcohol use, poor socioeconomic circumstances [3], and family history of suicide and mental illness [4]. An important risk factor related to suicide was mental illness, especially depression [5], and a lack of psychiatric resources was attributed to suicide deaths [6]. There is empirical evidence to establish the relationship between depression and suicide [7, 8]. Suicide rates among current and former psychiatric patients were found to range from four to six times the rate of the general population [9], and suicide rates remained high for many years after discharge from psychiatric admission [10]. In addition, depression was often associated with hopelessness and sleep disturbance [11], which increased suicide risk [12]. Predisposing factors to suicide during psychiatric treatment included more severe illness and a history of suicide attempts [13], which also predicted suicidal behavior after discharge from psychiatric inpatient admission [14].

A review of Western studies reported high rates of mental illness and comorbidity prior to suicide, namely, depression and alcoholism [15]. However, the frequency of this phenomenon was comparatively less in Asian studies [16]; for example, 63% [17] to 69.5% [18] of suicides in China had diagnosable mental illness. The percentage of suicides linked to depression was 40% in China [17] and 25% in India [19], as compared to 88% in Western countries [20]. Similarly, a recent review showed that diagnosis of depression in suicide attempters in Asia ranged from 22% to 59.7% [21]. It is unclear if the disparity is due to underdiagnosis of mental illness in Asian countries [22], possibly due to a comparative lack of psychological sophistication and psychosocial treatment resources and greater stigma attached to mental illness [23]. This phenomenon is concerning as accurate diagnosis could also facilitate access to appropriate treatment [11]. Interestingly, an ethnic inequality in diagnosis was also reported for Maori, Pacific, and Asian New Zealanders, who were more likely to be underdiagnosed with depression and anxiety disorders relative to European New Zealanders [24]. It remained unclear what contributed to this phenomenon. Similarly, there was indication of underdiagnosis of mental illness in Singaporean suicide attempters [25]. Singapore is a multiethnic society, with the main racial groups consisting of Chinese, Indians, and Malays, which offers a unique opportunity to study diagnosis of mental illness in a multiethnic sample of Asian suicide attempters. It was consistently found that Malays had lowest suicide rates, while Chinese had highest rates in suicide deaths, and Indians were overly represented in suicide attempts in Singapore [25, 26]. It would be of interest to study the diagnosis of mental illness in the multiethnic sample in Singaporean suicide attempters, as there is paucity of large-scale recent research in this area.

Over the past two decades, majority of suicide studies in Asia were conducted on suicide deaths [17, 19, 27, 28]. The current study on suicide attempts would be valuable in that it sought to enhance our understanding of relevant variables contributing to the diagnosis of mental illness in Asian suicide attempters in Singapore, with simultaneous consideration of psychosocial variables, e.g., risk and protective factors and variables related to the attempt. This could assist clinicians in succinct and accurate assessment, to derive an appropriate suicide management plan to prevent further suicide attempts [29]. Current international research embraces the concept of a mental illness continuum and cautions against an overarching definition of mental illness without considering the different contexts in which DSM (Diagnostic and Statistical Manual) diagnoses are used [30]. This exploratory study would add further insight into the evidence base for brief clinical assessment and diagnosis of mental illness in our local context.

As mental illness was among the strongest predictors of suicide [31], it is concerning that there might be underdiagnosis of mental illness in Asia [22]. Mental illness, such as depression, is associated with an elevated risk for suicide attempt [32]. The cultural formulation section of the Diagnostic and Statistical Manual, DSM [11], highlights consideration of contextual and psychosocial factors to inform diagnosis, management, and treatment of mental illness. To add to the complexity of culturally sensitive clinical assessment, it was suggested that Asian suicide attempters were more likely to self-report physical symptoms when interviewed by clinicians about their suicide attempts [29], which could be understood as cultural conceptualization of distress [32]. In a busy emergency department environment in Singapore, an efficient tool for diagnosis and assessment could enhance the provision of culturally appropriate clinical care for suicide attempters with mental illness; as mental health assessment and appropriate discharge planning are crucial components of comprehensive suicide prevention efforts [18]. Literature had underscored the importance of a biopsychosocial model in the assessment and treatment of mental illness, e.g., depression and suicidality following stressful life events [33]. Such a multidimensional framework could be useful in understanding assessment of mental illness in suicide attempters in Singapore [34]. However, there remained a paucity of recent large-scale research in Singapore to examine the variables clinicians used to diagnose mental illness and assess suicide risk for suicide attempters in Singapore. Recent large-scale studies on suicide attempters in Singapore indicated that gender and ethnicity contributed to suicide risk and protective factors and suicide lethality [25, 26], and psychosocial variables added to the complex clinical profile of suicide attempters in Singapore [29]. Considered together with our previous studies [25, 26, 29], the study of mental illness in suicide attempters in Singapore would add further insight, to inform our public mental health policies, and targeted suicide prevention strategies, especially for those at heightened risk for suicide and mental illness [35]. Mental illness increased the risk of suicide attempt; this dimension should be considered as an important therapeutic target to substantially advance our primary prevention efforts [36].

The current study aimed to examine the variables that contribute to clinicians' diagnosis of mental illness in a multiethnic sample of Asian suicide attempters in Singapore. Based on evidence in both Western and Asian studies, analysis would be conducted on the following variables, available as part of the Suicide Risk Assessment Form, SRAF, utilized by clinicians at the emergency department of the local hospital where this study took place. The SRAF was developed by the local hospital and utilized by clinicians for collection of clinically relevant variables, for assessment and diagnosis, and to devise an appropriate management plan, e.g., admission to psychiatric ward or outpatient follow up with a psychiatrist or medical social worker, or discharge. Psychometric properties were not available. Available psychosocial variables included the following risk factors: living alone [8], unemployment [29], financial problem [3], family history of suicide and psychopathology [37], physical illness [3], alcohol/drug use [38], and interpersonal conflict [29]. Protective factors included presence of dependents [39], emotional support [40], willingness to seek help [41], resolution of precipitants [42], religion [25], regret of the attempt [43], and positive future planning [44].

All available variables would be entered into the logistic regression. The available variables contained multidimensional variables well established in both Western and Asian studies, including the above-mentioned risk and protective factors as well as features of the attempt, e.g., planning, and precautions taken to hide the attempt [45, 46]. It was hypothesized that the model containing all available variables would be significant in distinguishing suicide attempters with and without mental illness.

## 2. Materials and Methods

### 2.1. Procedure

Ethics approval was obtained from the Domains-Specific Review Board of a large teaching hospital in Singapore and the Human Research Ethics Committee at James Cook University. This study was based on an archival retrospective review of deidentified hospital records of patients who were admitted for a suicide attempt from January 2004 to December 2006. Data were collected from hospital databases related to the suicide attempters who were admitted over the three-year period and this data set was the most comprehensive data set available from the hospital, as such assessment data were not collected prior to and following the stipulated period. Archival data were extracted from the Patient Psychiatric Assessment Form (PPAF). The PPAF included the Suicide Risk Assessment Form (SRAF), information about the current suicide attempt, as well as information about the suicide attempter, and risk and protective factors.

All cases of attempted suicide were assessed by clinicians in the emergency department under the supervision of a consultant psychiatrist, and the interview took approximately 20 minutes. This assessment was part of the protocol standard operating procedure for patients admitted following a medically treated suicide attempt. At the time of the evaluation, the medical officer made a formal psychiatric diagnosis based on DSM criteria. After the assessment, a management plan was recommended.

The inclusion criterion for the current study was patients who were admitted to the emergency department from January 2004 to December 2006 and were assessed by medical officers using the PPAF. There were a total of 671 cases of suicide attempts. Cases with missing data on key variables were removed from the data set (*n* = 209), resulting in a sample of 462 cases (70.6% females; 62.6% Chinese, 15.4% Malays, 16.0% Indians). Their age ranged from 12 to 86 (*M* = 29.37,* SD* = 12.89). The majority of them overdosed in the suicide attempt. Of the 462 cases, 25.1% of patients were assessed to meet DSM criteria for a formal psychiatric diagnosis at the time of evaluation. Of those diagnosed with mental illness, Table 1 shows the percentages for the respective diagnosis.

### 2.2. Materials

The Suicide Risk Assessment Form (SRAF) is a 2-page questionnaire, conducted as a semistructured interview by clinicians. The content of the assessment form included demographic information, details of the current attempt, mental status examination, and psychiatric diagnosis. Presence of prior planning, efforts to hide the suicide attempt, and usage of alcohol with the attempt were recorded on dichotomous scales. Risk factors were recorded on dichotomous scales and included lack of confidantes, living alone, unemployment, financial problem, mental illness or suicide in the family, alcohol or drug abuse, history of mental illness, interpersonal conflict, and poor coping. Protective factors were recorded on dichotomous scales and included presence of dependents, emotional support, willingness to seek help, resolution of precipitant, religion, regret, and positive future planning.

## 3. Results

The percentage of suicide attempters with risk factors, protective factors, and diagnosis of mental illness are presented in Table 1. In addition to risk and protective factors, features of the attempt, such as prior planning (11.0% Yes, 89.0% No), attempt to hide (30.3% Yes, 69.7% No), and place of suicide attempt (80.1% Home, 2.2% Workplace, 9.3% Public place, 2.8% Friend's house, .6% Public building) were also included in the analysis.

Direct logistic regression was performed to assess the impact of available variables, namely, risk factors, protective factors, and features of the suicide attempt on the likelihood that suicide attempters were diagnosed with mental illness. Logistic regression was used in similar studies for a large number of predictors [25, 26] and is typically used to develop a subset of variables useful for predicting the criterion, by eliminating superfluous variables. Our sample size is sufficiently large and representative for statistical regression [47]. The full model (see Table 2) containing all available predictors was statistically significant,* χ*2 (23,* N* = 462) = 83.40,* p* < .001, indicating that the model was able to distinguish between attempters with and without diagnosis of mental illness. The model as a whole explained between 16.5% (Cox and Snell *R*^2^) and 24.4% (Nagelkerke *R*^2^) of the variance in mental illness and correctly classified 79.0% of the cases. As shown in Table 3, only six of the independent variables made a unique statistically significant contribution to the model (unemployment, mental illness or suicide in family, alcohol or drug abuse, habitual poor coping, willing to seek help, and positive future planning). The strongest predictor of mental illness was mental illness or suicide in family, with an odds ratio of 2.75. This indicated that attempters who had mental illness or suicide in family were 2.75 times more likely to have a diagnosis of mental illness than those without mental illness, controlling for all other predictors in the model. The second strongest predictor was unemployment with an odds ratio of 2.43. This indicated that attempters who were unemployed were 2.43 times more likely to have diagnosis of mental illness. The third strongest predictor was willing to seek help, with an odds ratio of 2.28. This indicated that attempters who were willing to seek help were 2.28 times more likely to have diagnosis of mental illness. The fourth predictor was habitual poor coping, with an odds ratio of 2.20. This indicated that attempters with poor habitual coping were 2.20 times more likely to have diagnosis of mental illness. The fifth predictor was alcohol or drug abuse, with an odds ratio of 1.84. This indicated that attempters who abused alcohol or drugs were 1.84 times more likely to have diagnosis of mental illness. The last predictor was positive future planning, with an odds ratio of  .45. This indicated that attempters who had positive plans for immediate future were 45 times less likely to have diagnosis of mental illness.

## 4. Discussion

This study aimed to explore prediction of diagnosis of mental illness in suicide attempters in Singapore, using available variables. Three years of medical records of 462 suicide attempters were analyzed. As hypothesized, the full model with available risk and protective factors and features of the attempt was significant in predicting cases with and without diagnosis of mental illness and accurately classified 79% of suicide attempters with diagnosis of mental illness. The results offer preliminary evidence to support the current usage of brief assessment at the emergency department. Together with the mental status examination that was conducted routinely, the psychosocial assessment using a brief semistructured interview was supported by the results. The strongest predictor of diagnosis of mental illness among suicide attempters was mental illness or suicide in family, followed by unemployment, willingness to seek help, habitual poor coping, alcohol or drug abuse, and lastly, lack of positive future planning. The findings were consistent with previous literature associating suicide risk with mental illness or suicide in the family [37], unemployment, and alcohol or drug abuse [3]. Poor coping and lack of positive future planning could be associated with poor problem solving and reduced social support, in suicide attempters with mental illness [48].

The risk factors for mental illness in suicide attempters included psychosocial variables which often co-occurred in the psychiatric population [32], e.g., depressed individuals often suffered from impairment of planning and problem solving [48]. These variables might reflect the adverse impact of untreated debilitating mental illness which in turn have impact on employment and coping and also exacerbate the suicide risk. Patterns of co-occurring risk factors had been observed in suicides, e.g., mental illness with substance abuse, financial problems, relationship problems, recent crisis, and medical problems [3]; these multiple factors challenged the individuals' coping capacity and further increased the risk for repeated suicide attempts [29]. It is desirable to have multilevel modelling analysis to identify different layers of factors contributing to diagnosis of mental illness in suicide attempters, which take into account the social cultural environment, and other contextual factors, that might have impact on suicide attempters, e.g., socioeconomic status [49], and whether the attempters had received treatment and/or are currently receiving treatment for mental illness. However, there are constraints as such data were not collected. Future research could gather further information and employ multilevel modelling analysis to give further understanding into suicide attempters with mental illness. Further analysis could also employ in-depth analysis to explore how different diagnosis of mental illness might interact with various biopsychosocial risk and protective factors, as well as demographic factors in suicide risk analysis.

The result that suicide attempters more willing to seek help were more likely to be diagnosed with mental illness might seem counterintuitive, but consideration of literature on help-seeking behavior offers further insight into suicide attempts. Help-seeking behavior requires self-awareness of the problem, willingness to seek assistance, and social norms that encourage help-seeking [50]. Those willing to seek help might not have done so prior to the suicide attempt, due to stigma. Fear of stigma is prevalent in Asian societies [23]. Possible factors preventing suicide attempters from seeking help might include lack of knowledge of available services and difficulty in taking the initiative to seek help [51]. Such factors could be explored in future research through in-depth interviews.

It is noteworthy that in our sample, 25% were diagnosed with mental illness; this phenomenon is consistent with trends found in other Asian countries [17, 19]; this percentage is relatively low, as compared to Western countries [20]. A recent Asian study found that a risk factor for repeated suicide attempts included somatic complaints, which suggested the possibility that due to fear of stigma [25], Asian suicide attempters might be more willing to report physical symptoms rather than psychiatric concerns [29, 33], which could then lead to underdiagnosis of mental illness and exacerbate the risk of repeated suicide attempts. Such factors could be explored in future research through the inclusion of a standardized screening tool, e.g., PHQ-9 [52].

The current findings have implications for primary prevention, assessment, and case management of patients with mental illness and offer preliminary evidence to support multidisciplinary psychosocial assessment and interventions. Interventions could be more targeted, with greater service level collaborations to reach out to those with mental illness, e.g., mental health services, job agencies, counselling and drug and alcohol services. Consistent with previous research, preventive programs could be based on the detection of multidimensional psychosocial risk factors associated with both suicide and mental illness [53]. In addition, routine suicide assessment could be incorporated in case management of those with mental illness, and suicide assessments could also include routine screening of mental illness. Psychoeducation, screening, and interventions for mental health promotion could be encouraged in those with family history of mental illness or suicide. Counselling and crisis interventions could be targeted towards coping and positive future planning, in patients with mental illness. Such strategies focusing on problem solving skills are consistent with interventions suggested by previous Asian literature for suicide prevention [54].

Limitations to the study included the following: a lack of a control group limited how the data could be interpreted. Future research could compare data between suicide attempters with mental illness, suicide attempters without mental illness, and matched healthy controls for better interpretation of data. It is difficult to ascertain causality in cross sectional research. A longitudinal methodology could enhance our interpretation of the contribution of patterns of risk and protective factors across time. In addition, the contribution of ethnicity, migration status, and gender was unclear. There was indication from previous research these variables needed to be taken into account [25, 26]. However, the information on migration status was not collected in this study, future research could employ in-depth interviews to elicit the intricate interplay among these factors. Recent research has also suggested that suicide intent is an important variable [25]; although this is not the focus of the current study, intent could be a confounding variable, which could limit the generalizability of our results. Lastly, 209 cases were removed from the data set because of missing data on key variables, which might result in a biased sample for the current study. Similarly, this imposed a constraint on the generalizability of the results.

Other limitations of the study included the reliance on self-report and the brief nature of the assessment, as well as the usage of single items on dichotomous scales, which placed constraints on the depth of the information obtained. Future research could employ qualitative interviews to explore the relationships and processes underpinning risk and protective factors, features of the attempt, and other factors that might be relevant for understanding contributory factors towards mental illness in suicide attempters. The SRAF was developed by the local hospital for collection of clinically relevant variables, for assessment and diagnosis, and for devising an appropriate management plan, and psychometric properties were not available, which is another limitation. Future research could employ more comprehensive interviews and use standardized measures, e.g., Beck Suicide Ideation Scale for analysis of convergent validity and conduct the research with healthy controls for examination of discriminant validity [55]. Finally this study was based on an archival retrospective review of records of patients who were admitted for a suicide attempt from January 2004 to December 2006. Although this data set was the most comprehensive data set available from the hospital, as such assessment data were not collected prior to and following the stipulated period, the archival data were dated, and future research could endeavour to collect recent data. In addition, retrospective studies might be affected by recall bias that could significantly affect the reported findings. The strength of the study could be attributed to its large sample size, but in view of the above constraints, results need to be interpreted with caution.

## 5. Conclusion

In conclusion, the findings have implications for informing our efforts in assessment and primary prevention. The strongest predictor of diagnosis of mental illness among suicide attempters was mental illness or suicide in family, followed by unemployment, willingness to seek help, habitual poor coping, alcohol or drug abuse, and lastly lack of positive future planning. Such psychosocial factors could be included in routine psychiatric assessment to inform case management of patients with mental illness and underscore the importance of multidisciplinary collaboration. This study added to the current suicide literature and drew further focus to the importance of psychosocial assessment and interventions for those with mental illness.

## Figures and Tables

**Table 1:** Percentage of mental illness in suicide attempters (n=462).

	Percentage
**Mental illness**	25
(1) Depression	41
(2) Substance abuse	18
(3) Adjustment disorder	10
(4) Schizophrenia	8
(5) Borderline personality disorder	8
(6) Acute stress reaction	3
(7) Bipolar disorder	3
(8) Posttraumatic stress disorder	3
(9) Alcohol abuse	3

**Table 2:** Percentage of suicide attempters with risk factors, protective factors, and mental illness.

Predictors and criterion	Percentage	
	Yes	No
**Risk Factors**		
(1) Lack of confidantes	36.1	63.9
(2) Living alone	10.8	89.2
(3) Unemployment	16.9	83.1
(4) Serious financial problems	15.6	84.4
(5) Serious physical illness	4.8	95.2
(6) Mental illness/suicide in family	10.8	89.2
(7) Alcohol/drug abuse	18.2	81.8
(8) Ongoing interpersonal conflict	48.9	51.1
(9) Habitual poor coping	36.6	63.4
		
**Protective Factors**		
(1) Has dependents	59.1	40.9
(2) Emotional support	75.5	24.5
(3) Willing to seek help	69.6	30.1
(4) Resolution of precipitant	48.3	51.7
(5) Religion	35.1	64.9
(6) Expressed regret	80.1	19.9
(7) Positive future planning	74.9	25.1
		
**Criterion**		
(1) Mental illness	25.1	74.9

**Table 3:** Logistic regression predicting likelihood of attempters with diagnosis of mental illness.

Predictors	B	S.E.	Wald	*df*	*p*	Odds Ratio	95.0% CI for Odds Ratio	
							Lower	Upper
**Risk Factors**								
(1) Lack of confidantes	.38	.28	1.81	1	.18	1.45	.84	2.51
(2) Living alone	.40	.38	1.13	1	.29	1.49	.71	3.13
(3) Unemployment	.89	.31	8.30	1	.00	2.43	1.33	4.44
(4) Serious financial problems	-.29	.35	.71	1	.40	.75	.38	1.48
(5) Serious physical illness	.20	.56	.13	1	.72	1.22	.41	3.69
(6) Mental illness/suicide in family	1.01	.35	8.45	1	.00	2.75	1.39	5.42
(7) Alcohol/drug abuse	.61	.31	3.95	1	.05	1.84	1.00	3.35
(8) Ongoing interpersonal conflict	-.12	.25	.24	1	.63	.89	.54	1.44
(9) Habitual poor coping	.79	.26	9.07	1	.00	2.20	1.32	3.69
								
**Protective Factors**								
(1) Has dependents	-.14	.26	.29	1	.59	.87	.53	1.44
(2) Emotional support	-.26	.31	.71	1	.40	.77	.42	1.42
(3) Willing to seek help	.83	.30	7.52	1	.01	2.28	1.27	4.12
(4) Resolution of precipitant	-.34	.27	1.68	1	.20	.71	.42	1.19
(5) Religion	.16	.26	.37	1	.54	1.18	.70	1.97
(6) Expressed regret	-.17	.34	.25	1	.62	.84	.44	1.64
(7) Positive future planning	-.80	.29	7.57	1	.01	.45	.25	.79
								
**Features of the Attempt**								
(1) Prior planning	-.15	.40	.16	1	.69	.86	.40	1.84
(2) Attempt to hide	-.34	.28	1.52	1	.22	.71	.41	1.22
(3) Place of act			1.87	5	.87			
Home vs. workplace	.56	.83	.46	1	.50	1.75	.35	8.91
Home vs. public place	-.11	.41	.08	1	.78	.89	.40	2.01
Home vs. friend's house	.06	.82	.01	1	.95	1.06	.21	5.26
Home vs. public building	.25	1.27	.04	1	.85	1.28	.11	15.49
Home vs. others	-.75	.66	1.28	1	.26	.47	.13	1.73
								
Constant	-1.36	.48	7.89	1	.01	.26		

*Note.* Significant predictors are underlined. The *p* value for alcohol/drug abuse was rounded up from .047 to .05.

## Data Availability

The data used to support the findings of this study are included within the article.
